# Heatwaves, medications, and heat-related hospitalization in older Medicare beneficiaries with chronic conditions

**DOI:** 10.1371/journal.pone.0243665

**Published:** 2020-12-10

**Authors:** J. Bradley Layton, Wenhong Li, Jiacan Yuan, Joshua P. Gilman, Daniel B. Horton, Soko Setoguchi

**Affiliations:** 1 RTI Health Solutions, Research Triangle Park, Raleigh, North Carolina, United States of America; 2 Earth & Ocean Sciences, Nicholas School of the Environment, Duke University, Durham, North Carolina, United States of America; 3 Rutgers Robert Wood Johnson Medical School, New Brunswick, New Jersey, United States of America; 4 Department of Medicine, Boston University School of Medicine, Boston, Massachusetts, United States of America; 5 Department of Pediatrics, Rutgers Robert Wood Johnson Medical School, New Brunswick, New Jersey, United States of America; 6 Rutgers Center for Pharmacoepidemiology and Treatment Science, New Brunswick, New Jersey, United States of America; 7 Department of Medicine, Rutgers Robert Wood Johnson Medical School, New Brunswick, New Jersey, United States of America; Maastricht University Medical Center, NETHERLANDS

## Abstract

**Background:**

Heatwaves kill more people than floods, tornadoes, and earthquakes combined and disproportionally affect older persons and those with chronic conditions. Commonly used medications for chronic conditions, e.g., diuretics, antipsychotics disrupt thermoregulation or fluid/electrolyte balance and may sensitive patients to heat. However, the effect of heat-sensitizing medications and their interactions with heatwaves are not well-quantified. We evaluated effects of potentially heat-sensitizing medications in vulnerable older patients.

**Methods:**

US Medicare data were linked at the zip code level to climate data with surface air temperatures for June-August of 2007–2012. Patients were Medicare beneficiaries aged ≥65 years with chronic conditions including diabetes, dementia, and cardiovascular, lung, or kidney disease. Exposures were potentially heat-sensitizing medications including diuretics, anticholinergics, antipsychotics, beta blockers, stimulants, and anti-hypertensives. A heatwave was defined as ≥2 days above the 95^th^ percentile of historical zip code-specific surface air temperatures. We estimated associations of heat-sensitizing medications and heatwaves with heat-related hospitalization using self-controlled case series analysis.

**Results:**

We identified 9,721 patients with at least one chronic condition and heat-related hospitalization; 42.1% of these patients experienced a heatwave. Heatwaves were associated with an increase in heat-related hospitalizations ranging from 21% (95% CI: 7% to 38%) to 33% (95% CI: 14% to 55%) across medication classes. Several drug classes were associated with moderately elevated risk of heat-related hospitalization in the absence of heatwaves, with rate ratios ranging from 1.16 (95% CI: 1.00 to 1.35) to 1.37 (95% CI: 1.14 to 1.66). We did not observe meaningful synergistic interactions between heatwaves and medications.

**Conclusions:**

Older patients with chronic conditions may be at heightened risk for heat-related hospitalization due to the use of heat-sensitizing medications throughout the summer months, even in the absence of heatwaves. Further studies are needed to confirm these findings and also to understand the effect of milder and shorter heat exposure.

## Introduction

Over the last 50 years, an overall warming of the earth’s surface has resulted in both more frequent and severe extreme heat events [1,2], which are projected to increase in frequency, duration, and intensity in the coming decades [3,4]. Studies have reported that older adults with concomitant conditions, particularly including respiratory and cardiovascular disease, are at elevated risk for mortality and morbidity from heatwaves [5,6]. Older adults are especially vulnerable to extreme heat events, not only due to their age and high risk of disease burden, but also because of multiple comorbidities they may exhibit, resulting in the consumption of multiple medications concomitantly [7]. Polypharmacy (the use of multiple drugs concurrently) is very common in older adults, who make up only 13% of the US population but account for more than one third of all prescriptions dispensed [8,9]. The 2016 report on climate and health from the US Global Change Research Program recognized medications as an important risk factor for increased vulnerability in older adults [10].

Some classes of medications commonly used by older patients with chronic conditions may predispose these individuals to heat-related complications. These medications can sensitize a patient to heat by disrupting thermoregulatory responses that maintain core body temperature, either by interfering with cognitive processes or by directly disrupting autonomic mechanisms [11]. For instance, thermoregulation may be affected by numerous centrally-acting medications for neuropsychological disorders including antipsychotics, beta blockers, stimulants, and a broad array of medications with anticholinergic properties [11,12]. Dehydration with or without concurrent electrolyte disturbance may also contribute to thermoregulatory failure, and medications that suppress thirst [13] and disrupt fluid balance such as angiotensin converting enzyme (ACE) inhibitors and diuretics, are commonly used by older patients [11,14] with chronic conditions. Additionally, prior epidemiologic studies, mostly conducted in non-US samples or in the general population, support some of these hypotheses [15–19]. However, no formal epidemiologic study to date has examined the interaction between medication use and heatwaves in older adults, who are among the most vulnerable populations.

Based on underlying pharmacology and findings from previous studies [15–19], we selected several classes of potentially heat-sensitizing medications and evaluated their short-term associations with heat-related hospitalizations and potential interactions between medication exposure and heatwave events in older adults with chronic comorbidities.

## Materials and methods

We identified a cohort of older patients with chronic conditions who were discharged from the hospital and characterized their medication use after discharge from June 1 to August 31 of 2007–2012. As some of the medication classes of interest are key treatments for the relevant conditions, we did not compare medication-receiving patients to untreated patients, to avoid intractable confounding by disease severity, access to healthcare, and frailty [20]. Instead, we assessed if heat-related hospitalizations were more likely when a patient was using potentially heat-sensitizing medications compared to periods of non-use, accounting for periods of extreme heat during follow-up. We used self-controlled case series (SCCS) analysis [21], which estimates associations between transient exposures and acute outcomes while inherently controlling for time-invariant confounders such as underlying disease status.

The study was approved by the Institutional Review Board of the University of North Carolina at Chapel Hill. Individual patient consent was not required for this analysis of HIPAA-limited secondary data.

### Data source and population

We identified disease-based cohorts of patients aged ≥65 years among US Medicare beneficiaries with fee-for-service parts A, B, and D coverage who were discharged alive from the hospital with International Classification of Diseases 9^th^ Revision Clinical Modification (ICD-9-CM) codes for heart failure [22], myocardial infarction/acute coronary syndrome (MI/ACS), chronic obstructive pulmonary disease (COPD)/asthma, stroke [23], diabetes mellitus with chronic long-acting insulin use, dementia [24], or chronic kidney disease (CKD) [25] from June 1 to August 31 of each study year, 2007 to 2012; the hospital discharge date became the study index date. Each condition-specific cohort was identified separately, and an individual patient could contribute to multiple condition-specific cohorts. However, only a single hospitalization per person per condition was included; if a patient had multiple eligible hospitalizations for one condition, only the first within the study period was selected. Additionally, an overall cohort including all the conditions was identified, but patients with multiple disease-specific hospitalizations were included only once, at the earliest-occurring index hospitalization.

**Fig 1:**
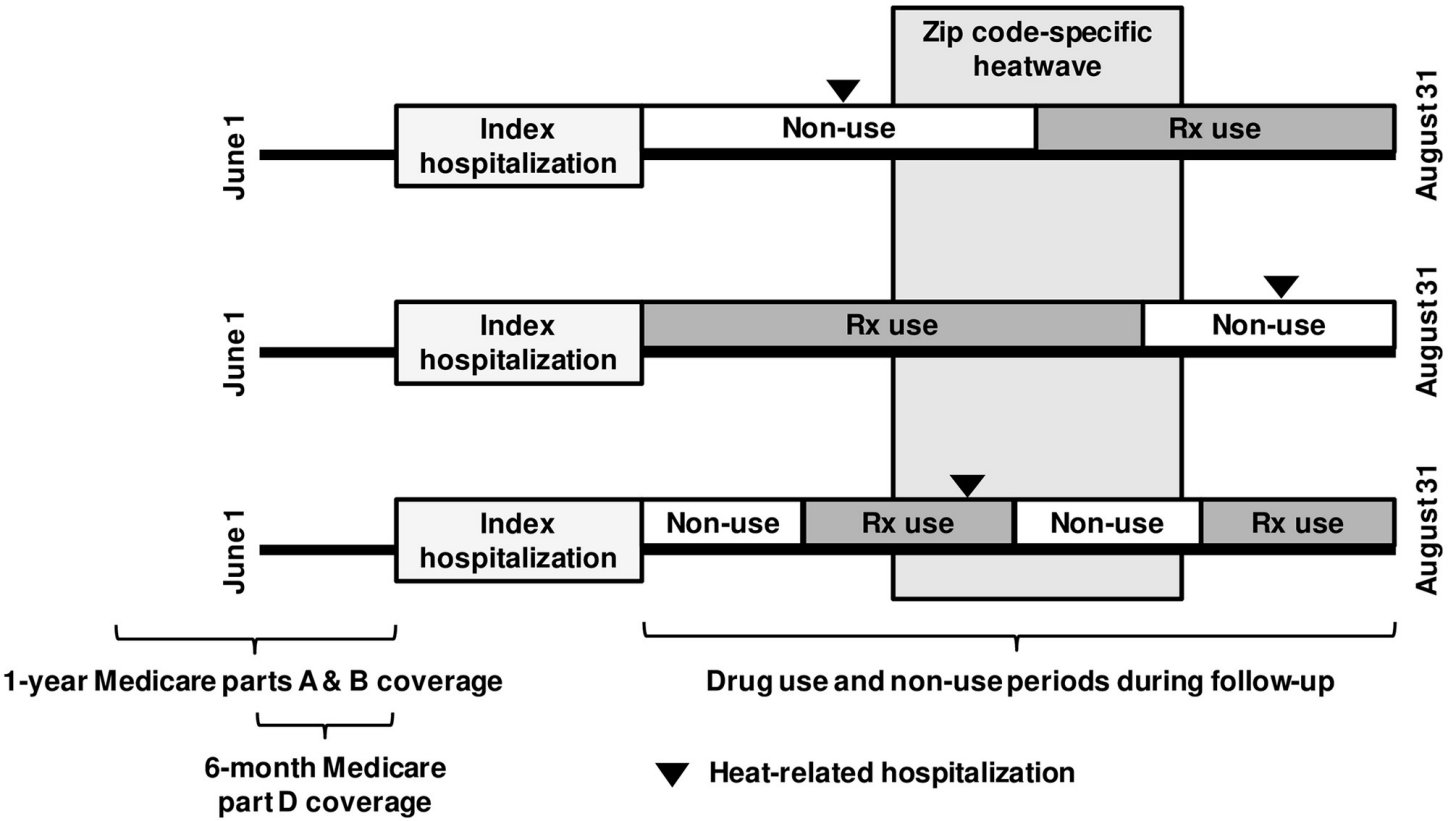
Schematic of three hypothetical patients from the same zip code with chronic conditions experiencing periods of medication use (Rx) and heatwaves during summer months.

We restricted our analyses to June-August in order to focus on the relatively short time periods when patients were exposed to higher temperatures and at greater risk of heat-related illness. Timing of most heatwave episodes tends to occur in late July and August, thus concentrating on these months is most appropriate [26,27]. We required that each patient have at least one year of continuous Medicare enrollment prior to the index hospitalization discharge. We followed patients from their index date to the earliest of August 31 of the given year, plan disenrollment, or mortality (Fig 1). To describe patient characteristics, we identified comorbidities from insurance claims in the year prior to the index hospital discharge.

### Outcome assessment

We identified heat-related hospitalizations during follow-up with ICD-9-CM diagnosis codes for heat-related illness [28–30] in any diagnosis position in the hospital record. The outcome definition included explicit diagnoses of heatstroke and other closely-related conditions associated with excessive heat [30–33], including effects of heat and light (992.0–992.9), excessive heat (E900.0, E900.9), dehydration (276.51), and exhaustion due to excessive exertion (994.5). In order to identify hospitalization for new-onset heat-related illness, transfers from another hospital or admissions for rehabilitation were not considered as a new hospital admission. We also conducted a sensitivity analysis using a broader outcome definition that also included hyperosmolality and/or hypernatremia (276.0).

### Heatwave exposure

The risk of heat-related illnesses is likely to be influenced by heatwaves—short-term elevations above normal temperatures [30–33]. Accordingly, we identified zip code-level heatwaves occurring during follow-up. With mean air temperatures and infrastructure (e.g., air conditioning) varying widely across the country, we focused on deviations above normal temperatures rather than on absolute temperatures. While there is no objective, uniform heatwave definition [34], we defined heatwaves as ≥2 consecutive days of maximum daily surface air temperatures (T_max_) above the 95^th^ percentile of historical zip code-specific normal temperatures (reference period 1949–2010) [35,36]. Due to differences in data availability, T_max_ data were obtained from the Meteorological Forcing Dataset for Land Surface Modeling for 2007–2010 [37] and from Daymet Daily Surface Weather Data for 2011–2012 [38]. We interpolated the gridded T_max_ to 43,191 zip codes across the United States. As heat effects may linger after the end of a defined heatwave, we added 14 days to the end of each heatwave period to capture potentially lagged heatwave-related events; additionally, if one heatwave’s grace period overlapped with the beginning of another heatwave event, we considered the two heatwaves as one continuous event.

### Medication exposure assessment

We evaluated each individual’s medication exposure using Medicare pharmacy dispensing data from the index hospitalization discharge through August 31 of that year. Medication classes were selected based on prior data and pharmacologic mechanisms and included ACE inhibitors or angiotensin II receptor blockers (ARBs), antipsychotics, beta blockers, loop diuretics, anticholinergic medications, and stimulants (S1 Table). Individual patients’ follow-up time was divided into periods of medication use and non-use based on individual patterns of medication initiation, refills, and discontinuation. The medication dispensing date was considered the beginning of use; if a refill or another medication in the same class was dispensed during the prescription period, the medication use period was extended by adding the new dispensed prescription’s day supply. If at the end of a prescription’s day supply, there had been no refill or new prescription, we allowed for a grace period of 50% of the last prescription’s day supply; if at the end of the grace period the patient had not received a refill or new prescription, the patient was considered to have discontinued the medication, and the use period ended. If the patient had a prescription prior to the index hospitalization, any remaining day supply at the time of hospitalization was carried over after discharge.

### Statistical analysis

Patient follow-up time from hospital discharge to August 31 of each year was divided into four exposure statuses based on medication and heatwave exposure (medication exposure, heatwave exposure, both, or neither). The rates of heat-related hospitalizations occurring during periods of medication use, heatwave exposure, or both combined were separately compared within individuals to rates during each patient’s periods of no medication or heatwave exposure (Fig 1). Analyses were performed separately for each medication class in all patients overall and separately within each comorbidity subgroup.

SCCS analyses are exposure-anchored, case-only designs restricted to only those patients experiencing the outcome. All of a patient’s follow-up time (both before and after the outcome) contributes to the analysis [39]. Standard SCCS analyses use conditional Poisson regression models to estimate the rate ratio (RR) and 95% confidence interval (CI) of the association between exposure and outcome by comparing the risk of the outcome during periods of exposure to that during periods of non-exposure within the same individual [40]. Because comparisons are made within an individual, the SCCS design implicitly controls for time-fixed characteristics during the study period (e.g., disease status, sex, race, genetics), but it is sensitive to time-varying factors that are associated with the outcome, and it requires that the probability of exposure not be affected by the occurrence of an outcome event [39]. As experiencing a major heat-related illness may lead to medication discontinuation, we used an adapted SCCS method for censored or curtailed data which assumes the counterfactual of no exposure after an event and uses unbiased estimating equations to account for changing post-event probability of exposure (e.g., reduced likelihood of future medication use after a major event or censoring due to death after the outcome) [41,42]. We performed the adapted SCCS to estimate the associations between current medication use and heat-related outcomes using available macros for SCCS analysis [43]. We also tested for evidence of statistical interaction between medication use and heatwaves in the overall cohort by including an interaction term between medication use and heatwave status. All analyses were performed using SAS 9.4 (SAS Institute, Cary, NC).

## Results

We identified 377,100 patients at hospital discharge having at least one of the comorbidities of interest (S3 Table). The final analytic dataset consisted of 9,721 (2.6%) cases who were subsequently hospitalized with a heat-related diagnosis, as SCCS analyses are restricted to only those who experience the outcome. The analytic sample was 63.9% female, 75.9% white, had a mean age of 80.4 years (standard deviation [SD] 8.3), and had a high burden of comorbidities (Table 1). The mean length of follow-up from discharge until censoring was 55.5 days (SD 23.2). Medication exposure was common, with 70.5% of patients using anticholinergic agents and 58.9% using beta blockers; there was little use of stimulants (Table 1). 42.1% of patients were exposed to at least one heatwave during follow-up (Table 1, S2 Table). Approximately 24–25% of the person-time under observation was classified as heatwave-exposed (Table 2). Death occurred in 1,275 (13.1%) patients during follow-up.

**Table 1:** Characteristics of the analytic cohort of patients with chronic conditions who subsequently experienced a heat-related hospitalization from June-August of 2007–2012.

Characteristic	All N = 9,721	CKD N = 4,205	Dementia N = 3,214	Heart failure N = 2,185	Diabetes mellitus N = 1,745	COPD N = 737	Myocardial infarction N = 608	Stroke N = 362
Age, mean (SD)	80.4 (8.3)	80.0 (8.2)	83.8 (7.5)	81.4 (8.4)	76.7 (7.6)	77.8 (7.6)	80.2 (8.1)	81.0 (8.4)
Sex, male	3,508 (36.1%)	1,740 (41.4%)	1,013 (31.5%)	779 (35.7%)	599 (34.3%)	263 (35.7%)	215 (35.4%)	114 (31.5%)
Race								
White	7,382 (75.9%)	3,157 (75.1%)	2,330 (72.5%)	1,744 (79.8%)	1,239 (71.0%)	617 (83.7%)	476 (78.3%)	256 (70.7%)
Black	1,623 (16.7%)	760 (18.1%)	640 (19.9%)	291 (13.3%)	338 (19.4%)	76 (10.3%)	78 (12.8%)	69 (19.1%)
Other	175 (1.8%)	83 (2.0%)	66 (2.1%)	35 (1.6%)	29 (1.7%)	NTSR	13 (2.1%)	13 (3.6%)
Asian	340 (3.5%)	123 (2.9%)	120 (3.7%)	74 (3.4%)	95 (5.4%)	20 (2.7%)	22 (3.6%)	13 (3.6%)
Hispanic	59 (0.6%)	25 (0.6%)	14 (0.4%)	13 (0.6%)	15 (0.9%)	NTSR	NTSR	NTSR
North American Native	122 (1.3%)	50 (1.2%)	33 (1.0%)	23 (1.1%)	29 (1.7%)	NTSR	14 (2.3%)	NTSR
Unknown	20 (0.2%)	NTSR	NTSR	NTSR	NTSR	NTSR	NTSR	NTSR
US Region								
Northeast	1,911 (19.7%)	797 (19.0%)	632 (19.7%)	487 (22.3%)	312 (17.9%)	140 (19.0%)	125 (20.6%)	64 (17.7%)
Midwest	2,278 (23.4%)	1,051 (25.0%)	676 (21.0%)	534 (24.4%)	432 (24.8%)	180 (24.4%)	141 (23.2%)	83 (22.9%)
South	4,241 (43.6%)	1,799 (42.8%)	1,506 (46.9%)	917 (42.0%)	769 (44.1%)	329 (44.6%)	254 (41.8%)	157 (43.4%)
West	1,268 (13.0%)	551 (13.1%)	393 (12.2%)	241 (11.0%)	225 (12.9%)	88 (11.9%)	85 (14.0%)	56 (15.5%)
Other	23 (0.2%)	NTSR	NTSR	NTSR	NTSR	NTSR	NTSR	NTSR
Comorbidities								
Atrial fibrillation	3,521 (36.2%)	1,631 (38.8%)	985 (30.6%)	1,261 (57.7%)	577 (33.1%)	248 (33.6%)	240 (39.5%)	130 (35.9%)
Anemia	6,414 (66.0%)	3,202 (76.1%)	2,059 (64.1%)	1,484 (67.9%)	1,187 (68.0%)	426 (57.8%)	376 (61.8%)	186 (51.4%)
Ischemic heart disease	6,379 (65.6%)	2,884 (68.6%)	1,794 (55.8%)	1,766 (80.8%)	1,229 (70.4%)	493 (66.9%)	608 (100.0%)	207 (57.2%)
Diabetes	5,516 (56.7%)	2,490 (59.2%)	1,517 (47.2%)	1,256 (57.5%)	1,745 (100%)	370 (50.2%)	343 (56.4%)	185 (51.1%)
COPD	5,003 (51.5%)	2,120 (50.4%)	1,388 (43.2%)	1,355 (62.0%)	880 (50.4%)	737 (100%)	321 (52.8%)	136 (37.6%)
Cancer	2,226 (22.9%)	1,161 (27.6%)	554 (17.2%)	424 (19.4%)	426 (24.4%)	154 (20.9%)	133 (21.9%)	55 (15.2%)
Heart failure	5,821 (59.9%)	2,616 (62.2%)	1,581 (49.2%)	2,185 (100%)	1,068 (61.2%)	475 (64.5%)	421 (69.2%)	155 (42.8%)
Myocardial infarction	2,049 (21.1%)	892 (21.2%)	449 (14.0%)	656 (30.0%)	380 (21.8%)	137 (18.6%)	608 (100.0%)	46 (12.7%)
Stroke	2,568 (26.4%)	1,017 (24.2%)	1,034 (32.2%)	490 (22.4%)	493 (28.3%)	125 (17.0%)	125 (20.6%)	362 (100.0%)
Dementia	4,254 (43.8%)	1,323 (31.5%)	3,214 (100%)	648 (29.7%)	567 (32.5%)	169 (22.9%)	163 (26.8%)	133 (36.7%)
CKD	5,546 (57.1%)	4,205 (100.0%)	1,315 (40.9%)	1,222 (55.9%)	1,076 (61.7%)	278 (37.7%)	273 (44.9%)	123 (34.0%)
Medication use								
ACE inhibitors	4,120 (42.4%)	1,607 (38.2%)	1,162 (36.2%)	1,020 (46.7%)	830 (47.6%)	273 (37.0%)	335 (55.1%)	156 (43.1%)
ARBs	1,757 (18.1%)	777 (18.5%)	427 (13.3%)	420 (19.2%)	369 (21.1%)	125 (17.0%)	102 (16.8%)	67 (18.5%)
Beta blockers	5,727 (58.9%)	2,552 (60.7%)	1,511 (47.0%)	1,566 (71.7%)	1,100 (63.0%)	342 (46.4%)	467 (76.8%)	197 (54.4%)
Loop diuretics	4,775 (49.1%)	2,100 (49.9%)	1,024 (31.9%)	1,704 (78.0%)	938 (53.8%)	367 (49.8%)	331 (54.4%)	115 (31.8%)
Anticholinergic agents	6,850 (70.5%)	2,902 (69.0%)	2,121 (66.0%)	1,631 (74.6%)	1,240 (71.1%)	497 (67.4%)	478 (78.6%)	231 (63.8%)
Antipsychotics	1,762 (18.1%)	556 (13.2%)	971 (30.2%)	240 (11.0%)	274 (15.7%)	85 (11.5%)	67 (11.0%)	46 (12.7%)
Stimulants	53 (0.5%)	25 (0.6%)	19 (0.6%)	NTSR	NTSR	NTSR	NTSR	NTSR
Heatwave exposure								
Experienced a heatwave	4,095 (42.1%)	1,769 (42.1%)	1,396 (43.4%)	919 (42.1%)	742 (42.5%)	324 (44.0%)	258 (42.4%)	151 (41.7%)
Number of heatwaves experienced, mean (SD)	0.9 (1.5)	1.0 (1.6)	1.0 (1.5)	0.9 (1.5)	1.0 (1.6)	1.0 (1.4)	1.0 (1.6)	0.9 (1.4)
Total days spent in a heatwave, mean (SD)	4.6 (10.4)	5.3 (11.5)	4.8 (10.4)	4.8 (10.7)	5.2 (11.8)	4.6 (9.9)	5.2 (11.3)	4.0 (8.5)

All numbers expressed as N (%) unless otherwise specified.

Abbreviations: ACE, angiotensin converting enzyme; ARB, angiotensin receptor blocker; CKD, chronic kidney disease; COPD, chronic obstructive pulmonary disease; NTSR, number too small to report; SD, standard deviation.

**Table 2:** Number of events (heat-related hospitalizations), total person-years, and drug and heatwave exposure distribution of person-time.

Cohort	Drug class	Number of events	Total person-years	% No drug, no heatwave	% Heatwave alone	% Drug alone	% Drug and heatwave
All	ACE inhibitors/ARBs	9,717	526,471	37.8	10.8	39.7	11.6
	Anticholinergic agents	9,718	526,391	26.4	7.7	51.1	14.7
	Antipsychotics	9,717	527,560	67.5	19.6	10.0	2.8
	Beta blockers	9,720	525,276	35.4	10.4	42.1	12.1
	Loop diuretics	9,720	524,943	43.3	12.2	34.2	10.4
	Stimulants	9,721	529,598	77.3	22.4	0.3	0.1
CKD	ACE inhibitors/ARBs	4,201	218,785	37.6	11.6	38.7	12.1
	Anticholinergic agents	4,205	218,757	25.9	8.3	50.5	15.4
	Antipsychotics	4,203	218,998	68.5	21.5	7.8	2.2
	Beta blockers	4,205	218,727	32.4	10.4	43.9	13.2
	Loop diuretics	4,205	218,639	40.9	12.3	35.5	11.4
	Stimulants	4,205	219,422	76.0	23.6	0.3	0.1
Dementia	ACE inhibitors/ARBs	3,212	147,848	42.7	12.1	34.4	10.9
	Anticholinergic agents	3,211	147,701	27.6	8.0	49.4	14.9
	Antipsychotics	3,212	147,623	57.2	17.3	19.8	5.7
	Beta blockers	3,213	147,609	42.7	12.9	34.3	10.0
	Loop diuretics	3,214	147,692	54.3	15.8	22.8	7.1
	Stimulants	3,214	148,066	76.6	22.8	0.4	0.1
Heart failure	ACE inhibitors/ARBs	2,184	118,475	33.3	10.1	43.3	13.3
	Anticholinergic agents	2,184	118,353	22.0	6.2	54.5	17.3
	Antipsychotics	2,185	118,538	70.2	21.4	6.3	2.0
	Beta blockers	2,184	118,282	24.4	7.5	52.2	16.0
	Loop diuretics	2,185	118,097	21.4	6.4	55.2	17.1
	Stimulants	2,185	118,689	76.3	23.4	0.3	0.0
Diabetes mellitus	ACE inhibitors/ARBs	1,744	97,655	29.7	8.5	48.4	13.4
	Anticholinergic agents	1,744	97,677	25.3	7.4	52.8	14.5
	Antipsychotics	1,744	97,717	69.3	19.7	8.7	2.2
	Beta blockers	1,744	97,642	31.2	9.1	46.9	12.8
	Loop diuretics	1,744	97,576	40.2	10.8	37.8	11.1
	Stimulants	1,745	97,957	77.7	21.8	0.4	0.1
Myocardial infarction	ACE inhibitors/ARBs	608	35,183	31.8	8.1	45.0	15.2
	Anticholinergic agents	608	35,139	19.7	6.3	57.0	17.0
	Antipsychotics	608	35,217	70.8	21.2	5.9	2.1
	Beta blockers	608	35,090	21.8	7.0	54.9	16.3
	Loop diuretics	607	35,057	40.8	12.1	35.9	11.2
	Stimulants	608	35,268	76.5	23.3	0.2	0.0
COPD	ACE inhibitors/ARBs	737	44,419	39.3	11.6	37.9	11.3
	Anticholinergic agents	737	44,404	28.6	8.7	48.5	14.1
	Antipsychotics	737	44,494	70.1	20.6	7.1	2.2
	Beta blockers	737	44,429	45.3	13.0	31.9	9.8
	Loop diuretics	737	44,343	41.7	12.3	35.5	10.5
	Stimulants	737	44,523	77.1	22.8	0.1	0.0
Stroke	ACE inhibitors/ARBs	362	18,665	35.1	10.2	44.4	10.3
	Anticholinergic agents	362	18,653	31.7	9.2	47.8	11.3
	Antipsychotics	362	18,671	71.1	18.5	8.4	2.0
	Beta blockers	362	18,662	41.9	10.7	37.7	9.7
	Loop diuretics	362	18,668	56.3	14.1	23.2	6.4
	Stimulants	362	18,715	79.4	20.5	0.1	0.0

Drug class refers to whether a patient had a prescription for a given drug at any point during the study period; person-time for “Drug alone” and “Drug and heatwave” refers to periods when the prescription was active.

Abbreviations: ACE, angiotensin converting enzyme; ARB, angiotensin receptor blocker; CKD, chronic kidney disease; COPD, chronic obstructive pulmonary disease.

Within individual comorbidity subgroups, the mean age of patients ranged from 76.7 years (SD 7.6) for diabetes mellitus to 83.8 years (SD 7.5) for dementia. The percentage of males varied from 31.5% for dementia and stroke to 41.4% for CKD, and the percentage of white patients ranged from 70.7% for stroke to 83.7% for MI. Heatwave exposure did not vary substantially across conditions (Table 2, S4 Table).

Of the heat-related hospitalizations observed during follow-up, 2,176 (22.4%) occurred during a heatwave period. The risk of experiencing a heat-related hospitalization was higher during heatwave periods, with RRs ranging from 1.21 (95% CI: 1.07 to 1.38) among ACE inhibitor/ARB users to 1.33 (95% CI: 1.14 to 1.55) among users of anticholinergic agents (Table 3). Comparing patients with each chronic condition, the associations between heatwave exposure and heat-related hospitalization were more pronounced in patients with diabetes mellitus.

**Table 3:** Risk of heat-related hospitalization by heatwaves, medications of interests, and their interactions.

		Rate ratio with 95% confidence interval			
Cohort	Drug class	Heatwave only	Drug only	Drug and heatwave	P-value for interaction
All	ACE inhibitors/ARBs	1.21 (1.07 to 1.38)	1.30 (1.13 to 1.50)	1.42 (1.21 to 1.67)	0.20
	Anticholinergic agents	1.33 (1.14 to 1.55)	1.16 (1.00 to 1.35)	1.26 (1.07 to 1.48)	0.02
	Antipsychotics	1.26 (1.14 to 1.39)	1.37 (1.14 to 1.66)	1.51 (1.20 to 1.91)	0.23
	Beta blockers	1.26 (1.10 to 1.45)	1.01 (0.88 to 1.17)	1.08 (0.92 to 1.27)	0.04
	Loop diuretics	1.25 (1.11 to 1.41)	1.32 (1.15 to 1.52)	1.52 (1.30 to 1.78)	0.33
	Stimulants	1.26 (1.15 to 1.39)	0.36 (0.13 to 0.97)	1.53 (0.54 to 4.34)	0.03
CKD	ACE inhibitors/ARBs	1.14 (0.93 to 1.41)	1.22 (0.92 to 1.62)	1.23 (0.90 to 1.68)	-
	Anticholinergic agents	1.21 (0.95 to 1.55)	0.88 (0.69 to 1.12)	0.90 (0.69 to 1.18)	-
	Antipsychotics	1.16 (0.99 to 1.35)	1.68 (1.18 to 2.40)	1.64 (1.07 to 2.51)	-
	Beta blockers	1.24 (0.98 to 1.55)	0.81 (0.64 to 1.04)	0.79 (0.60 to 1.04)	-
	Loop diuretics	1.07 (0.88 to 1.30)	1.15 (0.90 to 1.46)	1.30 (0.99 to 1.70)	-
	Stimulants	1.14 (0.98 to 1.32)	0.23 (0.06 to 0.93)	0.32 (0.03 to 3.57)	-
Dementia	ACE inhibitors/ARBs	1.14 (0.90 to 1.43)	1.06 (0.77 to 1.46)	0.96 (0.67 to 1.38)	-
	Anticholinergic agents	1.11 (0.83 to 1.48)	1.11 (0.76 to 1.63)	1.15 (0.77 to 1.72)	-
	Antipsychotics	1.12 (0.92 to 1.37)	1.07 (0.79 to 1.45)	1.01 (0.71 to 1.43)	-
	Beta blockers	1.12 (0.90 to 1.40)	0.89 (0.65 to 1.21)	0.91 (0.64 to 1.28)	-
	Loop diuretics	1.22 (1.00 to 1.50)	1.54 (1.06 to 2.24)	1.38 (0.92 to 2.07)	-
	Stimulants	1.13 (0.94 to 1.35)	0.14 (0.02 to 1.06)	6.70 (1.47 to 30.64)	-
Heart failure	ACE inhibitors/ARBs	0.99 (0.75 to 1.29)	1.23 (0.89 to 1.71)	1.18 (0.81 to 1.74)	-
	Anticholinergic agents	1.16 (0.79 to 1.71)	0.86 (0.60 to 1.25)	0.86 (0.58 to 1.28)	-
	Antipsychotics	1.08 (0.88 to 1.31)	0.84 (0.52 to 1.35)	0.62 (0.33 to 1.17)	-
	Beta blockers	1.04 (0.74 to 1.45)	0.68 (0.48 to 0.95)	0.69 (0.47 to 1.00)	-
	Loop diuretics	1.13 (0.82 to 1.56)	1.64 (1.20 to 2.23)	1.62 (1.17 to 2.25)	-
	Stimulants	1.13 (0.94 to 1.35)	0.14 (0.02 to 1.06)	6.70 (1.47 to 30.64)	-
Diabetes mellitus	ACE inhibitors/ARBs	1.49 (1.04 to 2.16)	1.22 (0.81 to 1.85)	1.42 (0.90 to 2.25)	-
	Anticholinergic agents	2.15 (1.44 to 3.21)	1.28 (0.86 to 1.90)	1.34 (0.88 to 2.04)	-
	Antipsychotics	1.64 (1.28 to 2.11)	1.83 (1.19 to 2.82)	1.82 (0.97 to 3.40)	-
	Beta blockers	1.76 (1.23 to 2.51)	0.95 (0.67 to 1.36)	1.00 (0.67 to 1.48)	-
	Loop diuretics	1.52 (1.10 to 2.11)	1.12 (0.78 to 1.63)	1.38 (0.91 to 2.09)	-
	Stimulants	1.13 (0.94 to 1.35)	0.14 (0.02 to 1.06)	6.70 (1.47 to 30.64)	-
Myocardial infarction*	ACE inhibitors/ARBs	1.12 (0.58 to 2.16)	1.31 (0.70 to 2.46)	1.79 (0.89 to 3.62)	-
	Anticholinergic agents	1.84 (0.89 to 3.79)	1.47 (0.83 to 2.59)	1.68 (0.90 to 3.13)	-
	Antipsychotics	1.53 (1.00 to 2.34)	1.39 (0.65 to 3.00)	1.90 (0.50 to 7.24)	-
	Beta blockers	1.27 (0.63 to 2.55)	1.38 (0.83 to 2.32)	1.63 (0.92 to 2.86)	-
	Loop diuretics	1.32 (0.77 to 2.27)	1.84 (1.13 to 3.00)	2.15 (1.22 to 3.78)	-
COPD*	ACE inhibitors/ARBs	0.92 (0.59 to 1.45)	1.18 (0.65 to 2.13)	1.49 (0.72 to 3.08)	-
	Anticholinergic agents	1.10 (0.67 to 1.81)	0.97 (0.54 to 1.76)	1.01 (0.53 to 1.92)	-
	Antipsychotics	1.04 (0.73 to 1.49)	0.71 (0.24 to 2.10)	1.06 (0.35 to 3.24)	-
	Beta blockers	1.09 (0.71 to 1.67)	0.54 (0.27 to 1.07)	0.58 (0.27 to 1.22)	-
	Loop diuretics	1.09 (0.70 to 1.69)	0.70 (0.43 to 1.14)	0.79 (0.45 to 1.39)	-
Stroke*	ACE inhibitors/ARBs	0.84 (0.42 to 1.68)	0.79 (0.34 to 1.83)	0.84 (0.31 to 2.31)	-
	Anticholinergic agents	1.42 (0.61 to 3.29)	1.90 (0.79 to 4.61)	2.19 (0.77 to 6.18)	-
	Antipsychotics	0.93 (0.53 to 1.64)	0.80 (0.24 to 2.63)	1.68 (0.44 to 6.40)	-
	Beta blockers	1.03 (0.49 to 2.19)	1.28 (0.53 to 3.05)	1.52 (0.59 to 3.91)	-
	Loop diuretics	1.02 (0.56 to 1.86)	0.68 (0.24 to 1.94)	0.75 (0.25 to 2.25)	-

Abbreviations: ACE, angiotensin converting enzyme; ARB, angiotensin receptor blocker; CKD, chronic kidney disease; COPD, chronic obstructive pulmonary disease.

*Model did not converge for stimulants due to small numbers.

Use of several medication classes (except for beta blockers and stimulants) was associated with heat-related hospitalization during the summer months among cases in the overall cohort, even during non-heatwave periods. RRs for positive associations ranged from 1.16 (95% CI: 1.00 to 1.35) for anticholinergic agents to 1.37 (95% CI: 1.14 to 1.66) for antipsychotics (Table 3). Among cases within the comorbidity-specific cohorts, use of loop diuretics was associated with heat-related hospitalization among patients with dementia (RR = 1.54, 95% CI: 1.06 to 2.24), heart failure (RR = 1.64, 95% CI: 1.20 to 2.23), and MI (RR = 1.84, 95% CI: 1.13 to 3.00) during non-heatwave periods, and use of antipsychotic medication was associated with heat-related hospitalization in patients with CKD (RR = 1.68, 95% CI: 1.18 to 2.40) and diabetes (RR = 1.83, 95% CI: 1.19 to 2.82) in the absence of heatwaves.

RRs for periods when patients were exposed to both medication and heatwaves were not meaningfully higher than those for the periods with heatwave only or medication-only estimates, suggesting an absence of positive synergistic effects between heatwaves and medications on heat-related hospitalization (Table 3).

In sensitivity analyses using broader heat-related hospitalization as an outcome, the number of heat-related hospitalizations increased by 1,523 (16%) but the medication and heatwave distribution in the cases was similar (S4 Table). Rate ratios for heat-related hospitalizations were similar but generally less pronounced using the broader definition for heat-related hospitalization (S5 Table).

## Discussion

In this SCCS analyses of older patients in the US with chronic conditions, heatwaves were associated with a 21% to 33% increased risk of heat-related hospitalization from June-August between 2007 and 2012. Use of medications that can potentially disrupt thermoregulatory responses which maintain core body temperature was frequent, ranging from 18% up to 71% except for stimulants (<1%). Exposure to these medications and exposure to heatwaves were both associated with increased risk of heat-related hospitalization. However, we found no meaningful, significant positive synergistic effects between heatwaves and most of the medications.

The effect sizes observed in our study are in a range consistent with those reported in previous studies of heatwaves and heat-related hospitalization. Bobb et al. linked 100% Medicare inpatient data and daily temperature data from the National Climatic Data Center and found that heatwave exposure was associated with increased risk of hospitalization for fluid and electrolyte disorders (relative risk = 1.18, 95% CI: 1.12–1.25) and heat stroke (relative risk = 2.54, 95% CI: 2.14–3.01) [33]. As our definition for heat-related hospitalization included heat stroke, dehydration and other heat-related events, we would expect our estimates to be within the range of these two estimates.

This national study evaluated older patients with chronic conditions from across the US treated in a variety of healthcare settings and experiencing a wide range of heat exposures. Findings showed that patients who experienced heat-related hospitalizations were more likely to experience them during periods of use of ACE inhibitors or ARBs, anticholinergic agents, antipsychotics, and loop diuretics. The observed increases in the risk of heat-related hospitalization among medication users are consistent with previous smaller studies of medication use and heat-related illness. However, studies supporting associations between medications and heat-related illness are limited to case reports [44], pharmacovigilance database reviews [16], prescription-only analyses [15], and case-control or case-only studies from hospitals and small samples in specific geographic areas [17–19].

We did not observe clinically meaningful synergistic effects between heatwave events and most of the medications. However, we did observe elevated rates of heat-related hospitalization associated with medication use throughout the warm summer months, even in the absence of prolonged extreme heat. Our heatwave definition did not capture extreme heat events lasting less than 2 days or below the 95^th^ percentile of historical temperatures, and it is possible that the medications under study may increase susceptibility to less extreme temperatures or shorter-lasting heat events in this vulnerable population. Results from the analyses of stimulant medications were based on fewer than 60 patients taking such medications in the overall cohort, with even lower numbers in the disease-specific cohorts, and therefore warrant further investigation in larger samples.

This national study of Medicare data benefits from the use of the SCCS design, which inherently controls for between-person confounding by time-invariant factors [39]. Our follow-up window was short (maximum of 3 months), so we anticipate minimal confounding by time-varying factors other than heatwave status, which was accounted for in the analysis.

Our study also has several limitations. Misclassification of drug exposures is possible, as we defined medication use from pharmacy dispensing data rather than direct observation of patient behavior. We may have overestimated or underestimated medication exposure due to non-adherence, early discontinuation, or our use of grace periods to define periods of continuous use. However, any exposure misclassification is likely to be non-differential and would thus lead to underestimation of the effect of medications on heat-related outcomes. We utilized a lag period in our heatwave definition to capture lagged events and concatenate adjacent heatwaves, but this may potentially introduce some misclassification of heatwave status. Our heatwave definition relied on zip code-level daily maximum temperatures at Medicare enrollees’ homes, though patient-level exposure to extreme heat may vary within zip codes. Patients may not be exposed to extreme heat at their home or current location due to travel or confinement to climate-controlled environments. For example, older patients with home health care or in a residential health facility may remain indoors and generally be shielded from outside temperatures, potentially illustrated by the generally reduced or null association of heatwave with heat-related hospitalizations among patients with stroke, who may be more frequently institutionalized or immobilized. However, the exact nature of patients’ heat exposure and home settings could not be inferred from our data. Accordingly, our findings may be subject to exposure misclassification that varies differentially by socioeconomic, household, or facility-level factors. However, as we employed a self-controlled case series design, non-time-varying socioeconomic factors are controlled by design, which would have greatly reduced the impact of this bias. In addition, climate exposures are influenced by many other factors including humidity and air quality, and additional analyses with more complex climate measurements are needed to expand on our findings. Our definition of heat-related hospitalization has been used in previous published studies but has not been validated in Medicare data; it likely captures only a subset of hospitalizations thought to result from heat effects, as the health effects of heat can also manifest as heart failure, syncope, shock or exacerbation of pre-existing conditions [31]. In addition, individuals who die of heat-related illness outside the hospital may not be accurately identified in this analysis, likely underestimating the effect of heat and medication on heat-related outcomes. The data for this study are from 2007–2012, and climate exposure levels may have changed since the end of the study period. Medication use patterns are also subject to secular trends, thus our findings should be confirmed with more recent data. Finally, we examined six medication classes and seven chronic conditions, and our analyses may thus have yielded some chance findings due to multiple comparisons.

While extreme heat events are known to have dangerous consequences for human health, this study showed that even in the absence of extreme heat, many commonly-used potentially heat-sensitizing medications increase the rate of heat-related hospitalization during the warm summer months in older patients with chronic conditions. The observed effects of medications on heat-related events may become more problematic in light of increasing average summer and night-time temperatures [45]. Older adults with serious chronic conditions similar to those we focused on in this study should protect themselves from summer heat, and providers caring for patients with these chronic conditions who are on one of the medication classes may consider monitoring these patients for signs of heat-related stress throughout the summer months. While further research is needed to confirm our findings, our study suggests that health care system improvements may be warranted, including changes to prescribing protocols and improvements to policies and practices for drug labeling, directions, and dissemination of information to the public during heatwave periods. With the “new normal” of COVID-19, those without air conditioning often cannot congregate in air-conditioned public spaces, which further increases the importance of informing clinicians and patients of the potential risk for heat-related adverse events in vulnerable populations. Our results were imprecise for less commonly used medications and for smaller subgroups, and our study did not take into account effects of other climate factors such as air pollution and humidity. High-resolution cohort studies including temperature, humidity and air pollution data in a larger population base can provide an opportunity to address these unanswered questions and expand our understanding of the role of medications under the changing climate.

## Supporting information

S1 Fig (DOCX)

S1 Table Medications included in each class of interest. (DOCX)

S2 Table Characteristics of US zip code-level heatwave events, defining heatwaves as ≥2 days greater than the 95^th^ percentile of daily maximum temperatures across a reference period from 1949–2010. (DOCX)

S3 Table Characteristics of all identified patients with chronic conditions discharged from June-August of 2007-2012. (DOCX)

S4 Table Number of events (broader heat-related hospitalizations), total person-years, and drug and heatwave exposure distribution of person-time. (DOCX)

S5 Table Risk of broader heat-related hospitalization by heatwaves, medications of interest, and their interactions. (DOCX)
