# Supplementary figures and images for: Heatwaves, medications, and heat-related hospitalization in older Medicare beneficiaries with chronic conditions
Source: PLoS One. 2020 Dec 10;15(12):e0243665. doi: 10.1371/journal.pone.0243665 (PMC7728169; doi:10.1371/journal.pone.0243665)

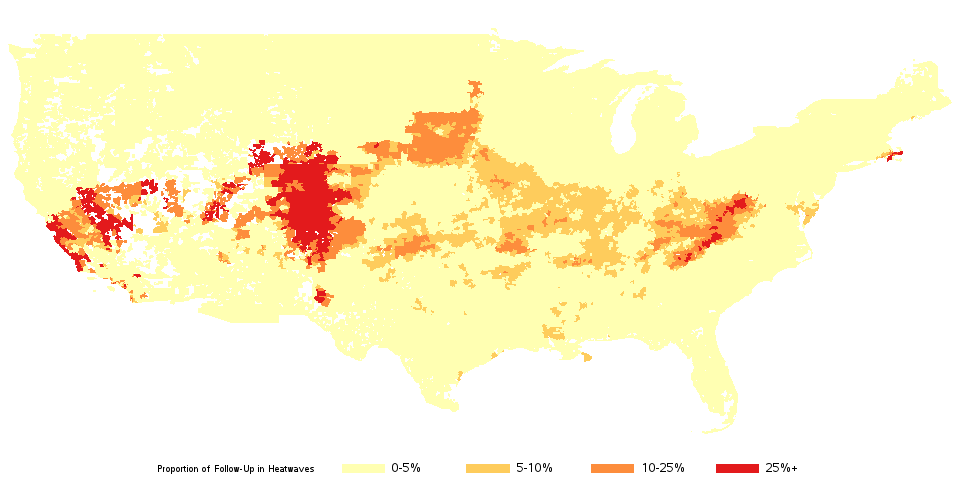

Supplement: S1 Fig — (DOCX) [file pone.0243665.s001.docx]
