# Supplementary material for: Heatwaves, medications, and heat-related hospitalization in older Medicare beneficiaries with chronic conditions
Source: PLoS One. 2020 Dec 10;15(12):e0243665. doi: 10.1371/journal.pone.0243665 (PMC7728169; doi:10.1371/journal.pone.0243665)
Supplement: S1 Table — (DOCX) [file pone.0243665.s002.docx]

**Table S1.** Medications included in each class of interest

| **Medication class** | **Included medications** | | |
| --- | --- | --- | --- |
| Angiotensin-converting-enzyme (ACE) inhibitors | Benazepril  Captopril  Enalapril  Enalaprilat | Fosinopril  Lisinopril  Moexipril  Perindopril | Quinapril  Ramipril  Trandolapril |
| Angiotensin II receptor blockers (ARBs) | Candesartan  Eprosartan  Irbesartan | Losartan  Valsartan  Olmesartan | Telmisartan |
| Anticholinergic medications | Acebutolol  Actophenazine  Ambenonium  Amfetamine  Amitriptyline  Anisotropine  Aripiprazole  Atenolol  Atomoxetine  Atropine  Belladonna alkaloids  Betaxolol  Bethanechol  Bisoprolol  Carteolol  Carvedilol  Cevimeline  Chlprothixene  Chlpromazine  Citalopram  Clidinium bromide  Clomipramine  Clozapine  Desvenlafaxine  Dexamfetamine  Dexmethylphenidate  Donepezil  Droperidol  Desipramine  Dicyclomine  Doxepin  Edrophonium  Ergotamine  Esmolol | Fencamfamin  Fenetylline  Fenozolone  Fluphenazine Galantamine  Glycopyrrolate  Haloperidol  Homatropine  Hexocyclium  Hyoscyamine  Ipratropium  Isocarboxazid  Isopropamide  Quetiapine  Risperidone  Thiidazine  Thiothixene  Trifluoperazine  Imipramine  Labetalol  Lisdexamfetamine Loxapine  Maprotiline  Memantine  Mepenzolate  Mesidazine  Metamfetamine  Methscopolamine Methantheline  Methylphenidate  Metoprolol  Mirtazapine  Modafinil  Molindone | Nadolol  Nebivolol  Neostigmine  Ntriptyline  Olanzapine  Oxyphencyclimine Paliperidone  Pemoline  Penbutolol  Pentaerythritol  Perphenazine Phenelzine  Physostigmine  Pilocarpine Pimozide  Pindolol Piperacetazine  Promazine  Prochlperazine Propantheline  Propranolol  Protriptyline  Pyridostigmin  Rivastigmine  Scopolamine  Sotalol  Tacrine  Timolol Tiotropium  Tranylcypromine  Trimipramine  Venlafaxine  Ziprasidone |
| Antipsychotics | Actophenazine  Aripiprazole  Chlpromazine  Chlprothixene  Clozapine  Droperidol  Fluphenazine  Haloperidol  Loxapine | Mesidazine  Molindone  Olanzapine  Paliperidone  Pentaerythritol  Perphenazine  Pimozide  Piperacetazine  Prochlperazine | Promazine  Quetiapine  Risperidone  Thiidazine  Thiothixene  Trifluoperazine  Ziprasidone |
| Beta blockers | Acebutolol  Atenolol  Betaxolol  Bisoprolol  Carteolol  Carvedilol | Esmolol  Labetalol  Metoprolol  Nadolol  Nebivolol  Propranolol | Penbutolol  Pindolol  Sotalol  Timolol |
| Loop diuretics | Bumetanide  Ethacrynate | Ethacrynic  Furosemide | Tsemide |
| Stimulants | Amfetamine  Dexamfetamine  Dexmethylphenidate  Fencamfamin | Fenetylline  Fenozolone  Lisdexamfetamine Metamfetamine | Methylphenidate  Modafinil  Pemoline |
