# Supplementary material for: Heatwaves, medications, and heat-related hospitalization in older Medicare beneficiaries with chronic conditions
Source: PLoS One. 2020 Dec 10;15(12):e0243665. doi: 10.1371/journal.pone.0243665 (PMC7728169; doi:10.1371/journal.pone.0243665)
Supplement: S2 Table — (DOCX) [file pone.0243665.s003.docx]

**Table S2.** Characteristics of US zip code-level heatwave events, defining heatwaves as ≥2 days greater than the 95^th^ percentile of daily maximum temperatures across a reference period from 1949-2010

| **Characteristic** | **Value** |
| --- | --- |
| Distinct zip codes experiencing at least one heatwave, N | 40,444 |
| Distinct zip code-level heatwave events, N | 367,083 |
| Mean heatwaves per zip code across all years (SD) | 9.1 (5.6) |
| Median length of heatwave, days (interquartile range) | 3 (2, 5) |
| Mean length of heatwave, days (SD) | 5.5 (7.9) |
| Total number of zip code-days in heatwave events | 2,022,570 |
| Number of zip code-level heatwave events by year (%) |  |
| 2007 | 64,945 (17.7) |
| 2008 | 31,641 (8.6) |
| 2009 | 27,024 (7.4) |
| 2010 | 63,005 (17.2) |
| 2011 | 96,937 (26.4) |
| 2012 | 83,531 (22.8) |
| Heatwave occurrence by month (%) |  |
| June | 97,644 (26.6) |
| July | 151,061 (41.2) |
| August | 118,378 (32.3) |

Abbreviations: SD, standard deviation
