# Supplementary material for: Heatwaves, medications, and heat-related hospitalization in older Medicare beneficiaries with chronic conditions
Source: PLoS One. 2020 Dec 10;15(12):e0243665. doi: 10.1371/journal.pone.0243665 (PMC7728169; doi:10.1371/journal.pone.0243665)
Supplement: S3 Table — (DOCX) [file pone.0243665.s004.docx]

**Table S3.** Characteristics of base cohorts of patients with chronic conditions for self-controlled case series analyses

| **Characteristic** | **All N=377,100** | **CKD N=156,562** | **Dementia N=107,679** | **Heart failure N=99,006** | **Diabetes mellitus N=72,697** | **Myocardial infarction N=44,481** | **COPD N=35,132** | **Stroke N=27,348** |
| --- | --- | --- | --- | --- | --- | --- | --- | --- |
| Age, mean (SD) | 79.6 (8.26) | 79.3 (8.16) | 83.5 (7.54) | 80.4 (8.22) | 76.0 (7.30) | 78.5 (8.15) | 76.7 (7.45) | 80.0 (8.11) |
| Sex, male | 141,127 (37.4%) | 67,729 (43.3%) | 32,417 (30.1%) | 36,276 (36.6%) | 26,636 (36.6%) | 18,869 (42.4%) | 12,647 (36.0%) | 9,697 (35.5%) |
| **Race** |  |  |  |  |  |  |  |  |
| White | 303,212 (80.4%) | 120,072 (76.7%) | 85,646 (79.5%) | 81,454 (82.3%) | 53,824 (74.0%) | 37,746 (84.9%) | 29,667 (84.4%) | 22,134 (80.9%) |
| Black | 48,605 (12.9%) | 25,059 (16.0%) | 15,146 (14.1%) | 11,841 (12.0%) | 12,415 (17.1%) | 3,871 (8.7%) | 3,462 (9.9%) | 3,314 (12.1%) |
| Asian | 6,961 (1.8%) | 3,409 (2.2%) | 1,707 (1.6%) | 1,497 (1.5%) | 1,370 (1.9%) | 815 (1.8%) | 529 (1.5%) | 657 (2.4%) |
| Hispanic | 11,646 (3.1%) | 4,920 (3.1%) | 3,581 (3.3%) | 2,664 (2.7%) | 3,433 (4.7%) | 1,248 (2.8%) | 969 (2.8%) | 748 (2.7%) |
| North American Native | 2,033 (0.5%) | 921 (0.6%) | 461 (0.4%) | 476 (0.5%) | 618 (0.9%) | 211 (0.5%) | 212 (0.6%) | 114 (0.4%) |
| Other | 4,029 (1.1%) | 1,915 (1.2%) | 939 (0.9%) | 908 (0.9%) | 931 (1.3%) | 518 (1.2%) | 257 (0.7%) | 334 (1.2%) |
| Unknown | 614 (0.2%) | 266 (0.2%) | 199 (0.2%) | 166 (0.2%) | 106 (0.1%) | 72 (0.2%) | 36 (0.1%) | 47 (0.2%) |
| **US Region** |  |  |  |  |  |  |  |  |
| Northeast | 73,826 (19.6%) | 30,219 (19.3%) | 21,933 (20.4%) | 20,813 (21.0%) | 13,422 (18.5%) | 8,969 (20.2%) | 6,852 (19.5%) | 5,049 (18.5%) |
| Midwest | 93,005 (24.7%) | 39,442 (25.2%) | 24,548 (22.8%) | 25,348 (25.6%) | 18,362 (25.3%) | 11,023 (24.8%) | 8,736 (24.9%) | 6,582 (24.1%) |
| South | 157,934 (41.9%) | 64,572 (41.2%) | 47,315 (43.9%) | 40,573 (41.0%) | 31,211 (42.9%) | 18,236 (41.0%) | 15,208 (43.3%) | 11,421 (41.8%) |
| West | 51,510 (13.7%) | 22,069 (14.1%) | 13,667 (12.7%) | 12,119 (12.2%) | 9,506 (13.1%) | 6,096 (13.7%) | 4,256 (12.1%) | 4,210 (15.4%) |
| Other | 825 (0.2%) | 260 (0.2%) | 216 (0.2%) | 153 (0.2%) | 196 (0.3%) | 157 (0.4%) | 80 (0.2%) | 86 (0.3%) |
| Comorbidities |  |  |  |  |  |  |  |  |
| Atrial fibrillation | 136,801 (36.3%) | 62,313 (39.8%) | 33,802 (31.4%) | 55,298 (55.9%) | 23,131 (31.8%) | 14,894 (33.5%) | 10,588 (30.1%) | 10,566 (38.6%) |
| Anemia | 220,138 (58.4%) | 115,509 (73.8%) | 63,353 (58.8%) | 60,263 (60.9%) | 45,115 (62.1%) | 21,638 (48.6%) | 16,700 (47.5%) | 11,306 (41.3%) |
| Ischemic heart disease | 247,429 (65.6%) | 109,344 (69.8%) | 57,827 (53.7%) | 79,165 (80.0%) | 51,832 (71.3%) | 44,481 (100.0%) | 21,264 (60.5%) | 14,725 (53.8%) |
| Diabetes | 203,677 (54.0%) | 95,139 (60.8%) | 46,701 (43.4%) | 55,037 (55.6%) | 72,694 (100%) | 22,402 (50.4%) | 16,334 (46.5%) | 12,332 (45.1%) |
| COPD | 183,142 (48.6%) | 76,931 (49.1%) | 44,231 (41.1%) | 58,499 (59.1%) | 35,129 (48.3%) | 19,334 (43.5%) | 35,132 (100%) | 9,420 (34.4%) |
| Cancer | 73,689 (19.5%) | 34,947 (22.3%) | 16,991 (15.8%) | 17,958 (18.1%) | 13,946 (19.2%) | 7,629 (17.2%) | 6,784 (19.3%) | 4,422 (16.2%) |
| Heart failure | 214,698 (56.9%) | 100,452 (64.2%) | 48,381 (44.9%) | 99,006 (100%) | 43,297 (59.6%) | 25,126 (56.5%) | 19,797 (56.4%) | 10,357 (37.9%) |
| Myocardial infarction | 89,068 (23.6%) | 35,477 (22.7%) | 14,516 (13.5%) | 28,345 (28.6%) | 16,245 (22.3%) | 44,481 (100%) | 5,224 (14.9%) | 3,558 (13.0%) |
| Stroke | 90,815 (24.1%) | 33,157 (21.2%) | 32,118 (29.8%) | 17,890 (18.1%) | 17,192 (23.6%) | 7,278 (16.4%) | 4,157 (11.8%) | 27,348 (100%) |
| Dementia | 133,691 (35.5%) | 37,525 (24.0%) | 107,679 (100%) | 21,954 (22.2%) | 17,050 (23.5%) | 7,899 (17.8%) | 5,909 (16.8%) | 7,375 (27.0%) |
| CKD | 186,965 (49.6%) | 156,562 (100%) | 34,595 (32.1%) | 49,248 (49.7%) | 40,298 (55.4%) | 15,912 (35.8%) | 9,672 (27.5%) | 7,622 (27.9%) |
| Medication use |  |  |  |  |  |  |  |  |
| ACE inhibitors | 144,905 (38.4%) | 56,630 (36.2%) | 33,522 (31.1%) | 42,832 (43.3%) | 32,184 (44.3%) | 20,629 (46.4%) | 10,928 (31.1%) | 10,200 (37.3%) |
| ARBs | 67,812 (18.0%) | 30,517 (19.5%) | 13,286 (12.3%) | 18,782 (19.0%) | 16,174 (22.2%) | 8,277 (18.6%) | 5,521 (15.7%) | 4,673 (17.1%) |
| Beta blockers | 215,782 (57.2%) | 96,124 (61.4%) | 47,622 (44.2%) | 69,406 (70.1%) | 44,772 (61.6%) | 32,872 (73.9%) | 14,040 (40.0%) | 14,206 (51.9%) |
| Loop diuretics | 165,911 (44.0%) | 77,326 (49.4%) | 32,213 (29.9%) | 72,207 (72.9%) | 37,163 (51.1%) | 17,036 (38.3%) | 15,113 (43.0%) | 6,658 (24.3%) |
| Anticholinergic agents | 259,277 (68.8%) | 106,808 (68.2%) | 71,280 (66.2%) | 73,041 (73.8%) | 49,778 (68.5%) | 33,696 (75.8%) | 23,408 (66.6%) | 16,679 (61.0%) |
| Antipsychotics | 44,976 (11.9%) | 12,905 (8.2%) | 28,112 (26.1%) | 6,792 (6.9%) | 7,086 (9.7%) | 2,541 (5.7%) | 2,661 (7.6%) | 2,014 (7.4%) |
| Stimulants | 1,559 (0.4%) | 531 (0.3%) | 640 (0.6%) | 312 (0.3%) | 328 (0.5%) | 116 (0.3%) | 121 (0.3%) | 125 (0.5%) |
| Heatwave exposure |  |  |  |  |  |  |  |  |
| Experienced a heatwave | 131,567 (34.9%) | 53,607 (34.2%) | 37,946 (35.2%) | 34,723 (35.1%) | 25,835 (35.5%) | 14,236 (32.0%) | 12,588 (35.8%) | 8,558 (31.3%) |
| Number of heatwaves experienced, mean (SD) | 0.7 (1.3) | 0.7 (1.4) | 0.7 (1.3) | 0.7 (1.3) | 0.8 (1.4) | 0.7 (1.3) | 0.8 (1.4) | 0.7 (1.3) |
| Total days spent in a heatwave, mean (SD) | 3.6 (9.2) | 3.7 (9.4) | 3.7 (9.3) | 3.6 (9.0) | 3.9 (9.6) | 3.3 (8.8) | 3.8 (9.4) | 3.1 (8.4) |
| Outcome |  |  |  |  |  |  |  |  |
| Heat-related hospitalization outcome^a^ | 9,721 (2.6%) | 4,205 (2.7%) | 3,214 (3.0%) | 2,185 (2.2%) | 1,745 (2.4%) | 608 (1.4%) | 737 (2.1%) | 362 (1.3%) |
| Heat-related hospitalization outcome (broad definition)^b^ | 11,244 (3.0%) | 4,881 (3.1%) | 3,826 (3.6%) | 2,547 (2.6%) | 2,048 (2.8%) | 698 (1.6%) | 830 (2.4%) | 437 (1.6%) |

All numbers expressed as N (%) unless otherwise specified.

^a^ Includes diagnosis codes for effects of heat and light (ICD-9-CM codes 992.0-992.9), excessive heat (E900.0, E900.9), dehydration (276.51), and exhaustion due to excessive exertion (994.5)

^b^ Includes all diagnosis codes for principal heat-related hospitalization outcome as well as hyperosmolality and/or hypernatremia (276.0)

Abbreviations: ACE, angiotensin converting enzyme; ARB, angiotensin receptor blocker; CKD, chronic kidney disease; COPD, chronic obstructive pulmonary disease; SD, standard deviation
