# Supplementary material for: Heatwaves, medications, and heat-related hospitalization in older Medicare beneficiaries with chronic conditions
Source: PLoS One. 2020 Dec 10;15(12):e0243665. doi: 10.1371/journal.pone.0243665 (PMC7728169; doi:10.1371/journal.pone.0243665)
Supplement: S4 Table — (DOCX) [file pone.0243665.s005.docx]

**Table S4.** Number of events (broader heat-related hospitalizations), total person-years, and drug and heatwave exposure distribution of person-time

| **Cohort** | **Drug** | **Number of events** | **Total person-years** | **% No drug, no heatwave** | **% Heatwave alone** | **% Drug alone** | **% Drug and heatwave** |
| --- | --- | --- | --- | --- | --- | --- | --- |
| All | ACE inhibitors/ARBs | 11,240 | 602,704 | 38.5 | 11.1 | 38.9 | 11.5 |
|  | Anticholinergic agents | 11,241 | 602,578 | 26.2 | 7.8 | 51.3 | 14.7 |
|  | Antipsychotics | 11,240 | 604,002 | 67.2 | 19.6 | 10.2 | 3.0 |
|  | Beta blockers | 11,243 | 601,304 | 35.1 | 10.5 | 42.3 | 12.1 |
|  | Loop diuretics | 11,242 | 600,596 | 42.6 | 12.1 | 34.8 | 10.5 |
|  | Stimulants | 11,244 | 606,200 | 77.2 | 22.4 | 0.3 | 0.1 |
| CKD | ACE inhibitors/ARBs | 4,877 | 250,139 | 38.4 | 11.9 | 37.9 | 11.7 |
|  | Anticholinergic agents | 4,881 | 250,086 | 25.5 | 8.3 | 50.9 | 15.3 |
|  | Antipsychotics | 4,879 | 250,370 | 68.5 | 21.3 | 7.9 | 2.3 |
|  | Beta blockers | 4,881 | 250,027 | 32.0 | 10.4 | 44.4 | 13.2 |
|  | Loop diuretics | 4,881 | 249,922 | 39.9 | 12.1 | 36.5 | 11.5 |
|  | Stimulants | 4,881 | 250,828 | 76.1 | 23.5 | 0.3 | 0.1 |
| Dementia | ACE inhibitors/ARBs | 3,824 | 173,627 | 43.6 | 12.6 | 33.4 | 10.4 |
|  | Anticholinergic agents | 3,823 | 173,483 | 27.5 | 8.2 | 49.5 | 14.8 |
|  | Antipsychotics | 3,824 | 173,394 | 57.0 | 17.2 | 20.0 | 5.8 |
|  | Beta blockers | 3,825 | 173,351 | 42.5 | 13.1 | 34.5 | 9.9 |
|  | Loop diuretics | 3,825 | 173,379 | 53.7 | 15.8 | 23.3 | 7.2 |
|  | Stimulants | 3,826 | 173,884 | 76.6 | 22.9 | 0.4 | 0.1 |
| Heart failure | ACE inhibitors/ARBs | 2,546 | 137,987 | 34.0 | 10.2 | 42.6 | 13.2 |
|  | Anticholinergic agents | 2,546 | 137,877 | 21.5 | 6.2 | 55.0 | 17.2 |
|  | Antipsychotics | 2,547 | 138,067 | 70.4 | 21.4 | 6.1 | 2.0 |
|  | Beta blockers | 2,546 | 137,783 | 24.1 | 7.5 | 52.4 | 16.0 |
|  | Loop diuretics | 2,546 | 137,494 | 20.7 | 6.3 | 55.9 | 17.1 |
|  | Stimulants | 2,547 | 138,248 | 76.3 | 23.4 | 0.2 | 0.0 |
| Diabetes mellitus | ACE inhibitors/ARBs | 2,047 | 113,962 | 30.6 | 9.0 | 47.0 | 13.3 |
|  | Anticholinergic agents | 2,047 | 113,966 | 24.8 | 7.5 | 52.8 | 14.9 |
|  | Antipsychotics | 2,047 | 114,039 | 68.8 | 20.1 | 8.8 | 2.3 |
|  | Beta blockers | 2,047 | 113,933 | 30.7 | 9.1 | 46.9 | 13.3 |
|  | Loop diuretics | 2,047 | 113,870 | 39.3 | 10.9 | 38.3 | 11.5 |
|  | Stimulants | 2,048 | 114,300 | 77.2 | 22.3 | 0.4 | 0.1 |
| Myocardial infarction | ACE inhibitors/ARBs | 698 | 39,626 | 32.0 | 7.9 | 44.6 | 15.5 |
|  | Anticholinergic agents | 698 | 39,586 | 20.4 | 6.5 | 56.2 | 16.9 |
|  | Antipsychotics | 698 | 39,669 | 71.1 | 21.5 | 5.5 | 1.9 |
|  | Beta blockers | 698 | 39,530 | 22.3 | 6.8 | 54.3 | 16.6 |
|  | Loop diuretics | 697 | 39,491 | 40.2 | 11.9 | 36.4 | 11.5 |
|  | Stimulants | 698 | 39,725 | 76.4 | 23.4 | 0.2 | 0.0 |
| COPD | ACE inhibitors/ARBs | 830 | 49,564 | 39.7 | 11.5 | 37.6 | 11.2 |
|  | Anticholinergic agents | 830 | 49,540 | 28.2 | 8.5 | 49.0 | 14.2 |
|  | Antipsychotics | 830 | 49,640 | 70.2 | 20.3 | 7.1 | 2.4 |
|  | Beta blockers | 830 | 49,561 | 44.7 | 12.9 | 32.6 | 9.9 |
|  | Loop diuretics | 830 | 49,465 | 41.7 | 11.9 | 35.6 | 10.9 |
|  | Stimulants | 830 | 49,673 | 77.2 | 22.7 | 0.0 | 0.0 |
| Stroke | ACE inhibitors/ARBs | 437 | 21,582 | 35.0 | 10.3 | 43.9 | 10.9 |
|  | Anticholinergic agents | 437 | 21,567 | 31.8 | 9.4 | 47.1 | 11.8 |
|  | Antipsychotics | 437 | 21,595 | 70.4 | 19.2 | 8.5 | 1.9 |
|  | Beta blockers | 437 | 21,572 | 40.1 | 11.5 | 38.7 | 9.6 |
|  | Loop diuretics | 437 | 21,585 | 55.7 | 14.9 | 23.2 | 6.3 |
|  | Stimulants | 437 | 21,640 | 78.6 | 21.2 | 0.2 | 0.0 |

Abbreviations: ACE, angiotensin converting enzyme; ARB, angiotensin receptor blocker; CKD, chronic kidney disease; COPD, chronic obstructive pulmonary disease
