# Supplementary material for: Heatwaves, medications, and heat-related hospitalization in older Medicare beneficiaries with chronic conditions
Source: PLoS One. 2020 Dec 10;15(12):e0243665. doi: 10.1371/journal.pone.0243665 (PMC7728169; doi:10.1371/journal.pone.0243665)
Supplement: S5 Table — (DOCX) [file pone.0243665.s006.docx]

**Table S5.** Risk of broader heat-related hospitalization by heatwaves, medications of interest, and their interactions

|  | | **Rate ratio with 95% confidence interval** | | |  |
| --- | --- | --- | --- | --- | --- |
| **Cohort** | **Drug** | **Heatwave only** | **Drug only** | **Drug and heatwave** | **P-value for interaction** |
| All | ACE inhibitors/ARBs | 1.20 (1.06 to 1.35) | 1.28 (1.12 to 1.46) | 1.41 (1.22 to 1.64) | 0.29 |
|  | Anticholinergic agents | 1.29 (1.12 to 1.50) | 1.13 (0.98 to 1.30) | 1.22 (1.05 to 1.43) | 0.04 |
|  | Antipsychotics | 1.25 (1.14 to 1.37) | 1.39 (1.16 to 1.66) | 1.49 (1.20 to 1.85) | 0.13 |
|  | Beta blockers | 1.24 (1.09 to 1.40) | 1.04 (0.91 to 1.18) | 1.12 (0.97 to 1.30) | 0.09 |
|  | Loop diuretics | 1.25 (1.12 to 1.40) | 1.31 (1.15 to 1.49) | 1.50 (1.29 to 1.73) | 0.23 |
|  | Stimulants | 1.25 (1.15 to 1.36) | 0.62 (0.26 to 1.50) | 1.61 (0.59 to 4.39) | 0.17 |
| CKD | ACE inhibitors/ARBs | 1.21 (1.00 to 1.46) | 1.19 (0.91 to 1.54) | 1.28 (0.96 to 1.70) | - |
|  | Anticholinergic agents | 1.28 (1.01 to 1.60) | 0.87 (0.69 to 1.10) | 0.94 (0.73 to 1.21) | - |
|  | Antipsychotics | 1.24 (1.07 to 1.44) | 1.59 (1.13 to 2.22) | 1.69 (1.14 to 2.51) | - |
|  | Beta blockers | 1.30 (1.06 to 1.60) | 0.82 (0.66 to 1.03) | 0.84 (0.65 to 1.08) | - |
|  | Loop diuretics | 1.16 (0.96 to 1.39) | 1.12 (0.90 to 1.40) | 1.31 (1.02 to 1.67) | - |
|  | Stimulants | 1.23 (1.07 to 1.42) | 0.45 (0.10 to 2.11) | 0.36 (0.04 to 3.47) | - |
| Dementia | ACE inhibitors/ARBs | 1.13 (0.92 to 1.39) | 1.08 (0.79 to 1.46) | 1.01 (0.72 to 1.43) | - |
|  | Anticholinergic agents | 1.14 (0.87 to 1.48) | 1.18 (0.82 to 1.71) | 1.24 (0.84 to 1.82) | - |
|  | Antipsychotics | 1.18 (0.98 to 1.41) | 1.12 (0.84 to 1.51) | 1.04 (0.75 to 1.46) | - |
|  | Beta blockers | 1.12 (0.92 to 1.38) | 1.02 (0.76 to 1.36) | 1.08 (0.79 to 1.49) | - |
|  | Loop diuretics | 1.24 (1.03 to 1.49) | 1.42 (1.01 to 1.98) | 1.27 (0.88 to 1.85) | - |
|  | Stimulants | 1.15 (0.98 to 1.35) | 0.60 (0.12 to 3.07) | 4.36 (0.58 to 32.99) | - |
| Heart failure | ACE inhibitors/ARBs | 0.99 (0.78 to 1.27) | 1.24 (0.91 to 1.69) | 1.23 (0.85 to 1.76) | - |
|  | Anticholinergic agents | 1.09 (0.76 to 1.56) | 0.87 (0.61 to 1.25) | 0.87 (0.60 to 1.27) | - |
|  | Antipsychotics | 1.07 (0.88 to 1.29) | 0.92 (0.58 to 1.45) | 0.76 (0.41 to 1.42) | - |
|  | Beta blockers | 1.02 (0.75 to 1.39) | 0.78 (0.57 to 1.07) | 0.79 (0.56 to 1.13) | - |
|  | Loop diuretics | 1.06 (0.78 to 1.44) | 1.50 (1.13 to 1.99) | 1.49 (1.09 to 2.02) | - |
|  | Stimulants | 1.15 (0.98 to 1.35) | 0.60 (0.12 to 3.07) | 4.36 (0.58 to 32.99) | - |
| Diabetes mellitus | ACE inhibitors/ARBs | 1.40 (1.01 to 1.94) | 1.31 (0.89 to 1.94) | 1.40 (0.92 to 2.14) | - |
|  | Anticholinergic agents | 1.90 (1.29 to 2.78) | 1.23 (0.86 to 1.77) | 1.25 (0.85 to 1.85) | - |
|  | Antipsychotics | 1.46 (1.16 to 1.84) | 1.85 (1.20 to 2.85) | 1.93 (1.09 to 3.41) | - |
|  | Beta blockers | 1.59 (1.14 to 2.20) | 1.08 (0.77 to 1.50) | 1.07 (0.75 to 1.54) | - |
|  | Loop diuretics | 1.45 (1.08 to 1.95) | 1.02 (0.72 to 1.45) | 1.15 (0.78 to 1.71) | - |
|  | Stimulants | 1.15 (0.98 to 1.35) | 0.60 (0.12 to 3.07) | 4.36 (0.58 to 32.99) | - |
| Myocardial infarction | ACE inhibitors/ARBs | 1.17 (0.64 to 2.15) | 1.41 (0.76 to 2.59) | 2.06 (1.04 to 4.09) | - |
|  | Anticholinergic agents | 1.67 (0.85 to 3.26) | 1.37 (0.78 to 2.41) | 1.74 (0.93 to 3.24) | - |
|  | Antipsychotics | 1.57 (1.05 to 2.34) | 1.35 (0.64 to 2.81) | 1.49 (0.45 to 4.92) | - |
|  | Beta blockers | 1.36 (0.71 to 2.61) | 1.43 (0.87 to 2.34) | 1.79 (1.03 to 3.11) | - |
|  | Loop diuretics | 1.39 (0.84 to 2.30) | 1.59 (0.99 to 2.56) | 1.98 (1.13 to 3.44) | - |
| COPD | ACE inhibitors/ARBs | 0.91 (0.59 to 1.40) | 1.19 (0.67 to 2.11) | 1.39 (0.70 to 2.75) | - |
|  | Anticholinergic agents | 1.00 (0.63 to 1.61) | 0.96 (0.54 to 1.68) | 0.96 (0.52 to 1.79) | - |
|  | Antipsychotics | 0.98 (0.69 to 1.39) | 0.95 (0.33 to 2.75) | 1.51 (0.54 to 4.22) | - |
|  | Beta blockers | 0.98 (0.65 to 1.48) | 0.58 (0.32 to 1.06) | 0.63 (0.32 to 1.24) | - |
|  | Loop diuretics | 1.01 (0.66 to 1.53) | 0.70 (0.45 to 1.10) | 0.76 (0.45 to 1.29) | - |
| Stroke | ACE inhibitors/ARBs | 0.84 (0.43 to 1.65) | 0.97 (0.42 to 2.27) | 0.86 (0.31 to 2.36) | - |
|  | Anticholinergic agents | 1.47 (0.70 to 3.11) | 2.57 (1.11 to 5.96) | 2.47 (0.94 to 6.49) | - |
|  | Antipsychotics | 0.90 (0.54 to 1.53) | 0.70 (0.21 to 2.31) | 1.19 (0.31 to 4.58) | - |
|  | Beta blockers | 1.02 (0.51 to 2.06) | 1.85 (0.82 to 4.17) | 1.97 (0.79 to 4.89) | - |
|  | Loop diuretics | 0.97 (0.56 to 1.70) | 0.93 (0.36 to 2.37) | 0.91 (0.32 to 2.63) | - |

Abbreviations: ACE, angiotensin converting enzyme; ARB, angiotensin receptor blocker; CKD, chronic kidney disease; COPD, chronic obstructive pulmonary disease
